# Ecology and evolution of pathogens in natural populations of Lepidoptera

**DOI:** 10.1111/eva.12328

**Published:** 2015-11-23

**Authors:** Judith H. Myers, Jenny S. Cory

**Affiliations:** ^1^ Department of ZoologyUniversity of British ColumbiaVancouverBCCanada; ^2^ Department of Biological SciencesSimon Fraser UniversityBurnabyBCCanada

**Keywords:** disease ecology, disease transmission, forest Lepidoptera, insect pathogens, migration, population regulation, tritrophic interactions, virulence

## Abstract

Pathogens are ubiquitous in insect populations and yet few studies examine their dynamics and impacts on host populations. We discuss four lepidopteran systems and explore their contributions to disease ecology and evolution. More specifically, we elucidate the role of pathogens in insect population dynamics. For three species, western tent caterpillars, African armyworm and introduced populations of gypsy moth, infection by nucleopolyhedrovirus (NPV) clearly regulates host populations or reduces their outbreaks. Transmission of NPV is largely horizontal although low levels of vertical transmission occur, and high levels of covert infection in some cases suggest that the virus can persist in a nonsymptomatic form. The prevalence of a mostly vertically transmitted protozoan parasite, *Ophryocystis elektroscirrha*, in monarch butterflies is intimately related to their migratory behaviour that culls highly infected individuals. Virulence and transmission are positively related among genotypes of this parasite. These systems clearly demonstrate that the interactions between insects and pathogens are highly context dependent. Not only is the outcome a consequence of changes in density and genetic diversity: environmental factors, particularly diet, can have strong impacts on virulence, transmission and host resistance or tolerance. What maintains the high level of host and pathogen diversity in these systems, however, remains a question.

## Introduction

It has long been known that insects support a wide diversity of pathogens, including fungi, bacteria, protozoans and DNA and RNA viruses (Fuxa and Tanada 1987). The field of insect pathology can be traced back to China in 2700 BC and the study of the pathogens of silkworms (Tanada and Kaya 1993). Early work focussed on diseases of economically important insects, but it was also recognized in the 1800s that pathogens might have potential for controlling insect pests. The development of insect pathogens for biological control agents continues (Lacey et al. 2001; Hajek 2004). Most research concerns pathogens that are highly pathogenic and have distinctive symptoms that make them easy to identify. However, recent developments in molecular and genomic techniques have made the identification and study of pathogens, including those without visible symptoms, considerably easier (Vega and Kaya 2012). Given the long history of study, it is perhaps surprising that the impacts of pathogens on insect population densities in the field are little studied as compared to the influences of predators and parasitoids (Roy et al. 2009).

The models of Anderson and May (1981) initially explored the potential for pathogens to regulate insect population densities, and these models have continued to be refined and developed (overview in Elderd 2013). In addition, insect pathogens are increasingly being used to address fundamental questions relating to disease ecology, ecological immunology and pathogen evolution (Hawley and Altizer 2011; Schmid‐Hempel 2011). However, the link between empirical studies of invertebrate pathology and disease ecology remains weak, and many theories and models of host–pathogen interactions remain untested. Long‐term studies of pathogens in natural insect populations are rare.

The dynamics of pathogen–host interactions in insects are determined primarily by host and pathogen density, pathogen transmission and pathogen virulence (impact on infected individuals ranging from slightly debilitating to lethal). The availability of insect hosts varies widely over time through seasonal cycles (overwintering in temperate climates), population cycles, as demonstrated by a number of forest Lepidoptera (Myers and Cory 2013), and migratory behaviour as occurs in a number of Lepidoptera that move annually to seasonally available habitats from overwintering areas. This variation in host abundance contributes to the value of insect studies for informing important questions in disease ecology related to the impact of pathogens on host densities, the importance of genetic variation, selection on host resistance, the evolution of virulence and the role of different transmission strategies on pathogen persistence.

Here, we analyse four well‐studied systems: the western tent caterpillar, *Malacosoma californicum pluviale*, the African armyworm, *Spodoptera exempta,* the gypsy moth, *Lymantria dispar*, and the monarch butterfly, *Danaus plexippus*. These species differ in their ecologies and their dynamics. The temperate tent caterpillars have cyclic population dynamics, and the tropical African armyworm has outbreak dynamics with multigenerational, long‐distance migrations. Gypsy moth is an introduced species to North America that has sporadic outbreaks and is still spreading. Monarch butterflies migrate annually in eastern and western North America from overwintering sites to reproductive sites and back. Three of the species, gypsy moth, armyworm and tent caterpillars are infected at high density by nucleopolyhedroviruses (NPV) (Baculoviridae), which are highly pathogenic, and primarily horizontally transmitted. Monarch butterflies are infected by a neogregarine protozoan parasite, *Ophryocystis elektroscirrha,* which is mainly transmitted vertically from mothers to offspring through spores on the eggs. In addition, gypsy moth is infected by an introduced fungus, *Entomophaga maimaiga,* which is horizontally transmitted and can cause high levels of mortality.

Our goal is to review lepidopteran–pathogen dynamics and examine the insights that have been gained from these four particularly comprehensive studies involving insect pathogens. In particular, we focus on pathogen transmission, the role of environmental factors in modulating the host–pathogen relationship and the impacts of the pathogens on host population dynamics. We consider whether pathogens have a role in determining insect population dynamics or whether there are traits that contribute to their importance in determining host densities and their persistence. These examples reflect our long term fascination with the role of disease as an influence on host population dynamics and the evolutionary changes and constraints that mold these interactions (Box 1).

Box 1 Judith Myers – My career has been serendipitous. My first experience in ecology came from the luck of getting a scholarship to do a marine ecology course at Woods Hole Laboratories (Mary Jane West‐Eberhard was my roommate there). Next I went to Trinidad to study butterfly courtship because I babysat the son of famous biologists Lincoln and Jane Brower. This led to a M.Sc. degree at Tufts University and the discovery of a gregarine parasite of monarch butterflies, part of the story in this article. When a certain famous biologist told me that women do not use their Ph.Ds but just get married and have babies, I decided to move to another project for my PhD at Indiana University. I worked on with Charles Krebs, who has continued as a mentor through my career. Here, I became fascinated with population cycles, another part of the following article. A Miller postdoc at Berkeley in 1970–1972 allowed me to see political unrest up close following the bombing of Cambodia, including the smell of tear gas. In 1972, universities were much more interested in hiring women than they had been in 1970. I chose a position at UBC and went from working on small mammals back to working with plants and insects. As it happened, Jamie Smith was hired by the Zoology Department the next year. He was single, I was single; who could believe. Our first sabbatical to Australia resulted in an additional project; we had a daughter Isla (now a Chancellor's Fellow at Edinburgh University and doing her research in Arctic and alpine areas in the Yukon). Our son was born 3 years later and I was the first woman in the Faculty of Science to take maternity leave. In 1986–1987, Jamie and I went to Oxford on sabbatical leave. It was easy for Jamie to arrange a connection there as that is where he had done his DPhil. But Sir Richard Southwood, the head of the Zoology Department at the time, told me there was no office space although I could ‘sit on my husband's knee’. This resulted in my hanging out at the nearby NERC Institute of Virology where Jenny Cory taught me about insect viruses. We have collaborated ever since. In the early 1990s, I became an associate dean in the Faculty of Science to promote women. I fought for mentoring, policies for hiring that were more likely to include women, and spoke out at promotion and tenure meetings when the records of women were being judged on a different standard than men; ‘she hasn't done enough yet or it is all her advisor's work’. My recommendations to young women are as follows: Don't readily take ‘no’ for an answer, hire a cleaning person as soon as you can, marry someone as smart or smarter than yourself who can cook, don't be afraid to wait until after you get tenure to have children, enjoy what you do and keep smiling.

Jenny Cory – As the first in my family to go to University my choice of place and subject was rather arbitrary. I opted for Zoology at The University of Sheffield. My main criterion was being far enough away from my London home, not because I wanted to escape but because I wanted to reduce the temptation to return. There I became fascinated with Ecology, mainly through the course being run by a then new lecturer, Tim Birkhead, and several wet and windy field trips to the Welsh Coast. I did an honours project on bird behaviour and envisaged myself as a vertebrate behavioural ecologist. I thought that I had a PhD lined up in this area, but it fell through. I remember telling my head of department that there were many topics that interested me, but I did not want to work with insects. Fast forward a few months and I was starting a DPhil. at Oxford University on insect predators and I have worked with insects ever since. If I had known, then what I do now I might have planned my career more carefully: working in the right laboratories and on the more high profile systems; I knew nothing about academic life and had no one to advise me. However, I have always found that if things did not work out quite as planned, something else would turn up and perhaps turn out to be even better. Oxford was a great experience: my supervisor Martin Speight was very supportive and gave me a free hand to do more or less what I wanted. And I thoroughly enjoyed the college experience and the wide diversity of friends that I made there. After toying with the idea of going somewhere more exotic, I was offered a permanent job at the NERC IOV that was too good to refuse; I stayed for 18 years. This meant learning a whole new area (insect virus ecology and microbial control) and adapting to the different priorities associated with working in a government laboratory. My career was impacted by several interesting characters at IOV. Philip Entwistle was a wealth of knowledge about insect virus ecology and in many ways was ahead of his time in terms of disease ecology. Here, I developed my passion for insect disease ecology. My career aims have been to move this topic into mainstream ecology and to introduce more ecological thinking into the application of microbial insecticides. A second influence was David Bishop, the Institute's director, who taught me how to argue for what I believed was worth doing, gave me many opportunities for thinking on my feet and challenged me to push myself further. I had never quite believed the stories about women with equivalent or better CVs being judged more negatively than men, but I saw it time and time again on promotion panels. Clearly having women on panels and committees is crucial. Meeting Judith Myers was one of the highlights of my time at NERC, and she has been the most important mentor in my career. In 1988, she provided funding for me to give an invited presentation at the International Congress of Entomology in Vancouver. This initiated a deep affection for British Columbia, cemented by a sabbatical at UBC in the late 1990s. I immigrated to Canada in 2005 to take a tier 1 CRC at Algoma University and the ‘Bug Lab’ in Sault Ste. Marie, Ontario. Living in northern Ontario, ‘real Canada’, was a new experience; long winters, isolation, small planes to Toronto, but I was able to play a role in building the Biology programme which assisted in the transition of Algoma to an independent university. In 2008, I became Chair in Biological Control and director of the Masters in Pest Management Program at Simon Fraser University in Burnaby, BC. I think that being a woman in science has improved markedly in recent years, but the increasingly competitive environment and the trend towards assessment by media acclaim is going to mean that mentoring and networking will be increasingly important for maintaining a diverse scientific culture. I am still working on hiring a cleaner….

This paper reflects our interests in the ecology and evolution of insect diseases, particularly those of Lepidoptera. This is not a comprehensive review but covers some examples that we feel are particularly interesting including our own projects on tent caterpillars and African armyworm, and the fascinating studies initiated by colleagues Ann Hajek and Sonia Altizer.

## Western tent caterpillar

Western tent caterpillars are native to coastal areas of western North America from California to British Columbia. They have one generation a year and overwinter as pharate larvae in egg masses laid in late June on a variety of host trees including red alder, *Alnus rubra*, apple trees, *Malus* spp. wild rose, *Rosa nutkana*, and wild cherry, *Prunus emarginata*. Larvae hatch in early April, remain together as families and form silken tents that they use for protection, and as a platform for basking in the sun. Larvae feed gregariously until late instars when they leave the tent to feed and pupate. Adults emerge in mid‐ to late June. Females mate very shortly after emergence and lay all of their eggs in a single mass of approximately 150 to 250 eggs. Some females, however, must fly considerable distances as populations of tent caterpillars show little genetic population structure (Franklin et al. 2014).

We have monitored populations of tent caterpillars by counting tents over the last 40 years on the Southern Gulf Islands and the lower mainland of British Columbia Canada. Typical of a number of forest Lepidoptera, their dynamics are cyclic with an 8‐ to 11‐year periodicity (Myers and Cory 2013). Since 1991, levels of infection by a nucleopolyhedrovirus (McplNPV) have also been recorded (Cory and Myers 2009). In addition, experiments have been used to gain an understanding of the ecology and evolution of this insect–pathogen system including transmission, food plant effects, fecundity changes and population regulation.

**Figure 1:**
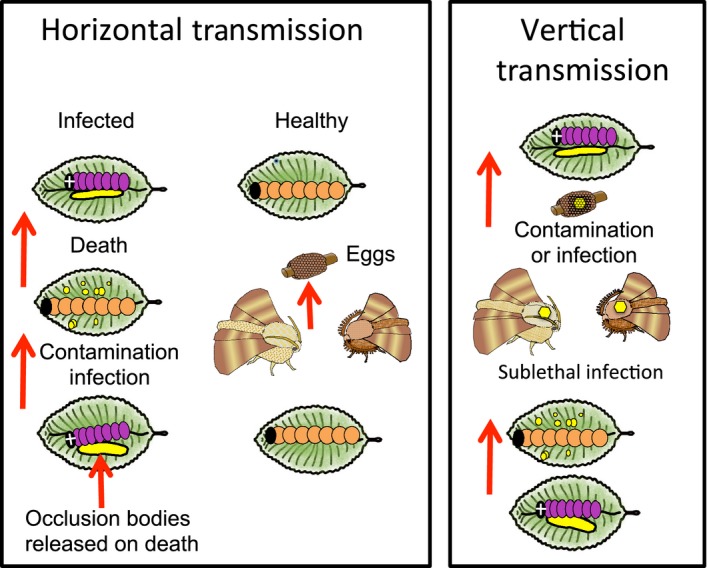
Transmission of baculoviruses can be horizontal among individuals through environmental contamination, or vertical from parents to offspring on or in the eggs. Upon death of an infected larva, occlusion bodies are released and other larvae become infected if they ingest the occlusion bodies on contaminated foliage. Vertical transmission can occur if larvae consume occlusion bodies (small yellow dots) but pupate before death leading to sublethally infected adults (indicated by larger yellow dot). Adults sublethally infected as larvae might transmit virus to their offspring either on or in the eggs. This can lead to an active infection killing the offspring or potentially a covert infection that is passed on to offspring that will survive.

**Figure 2:**
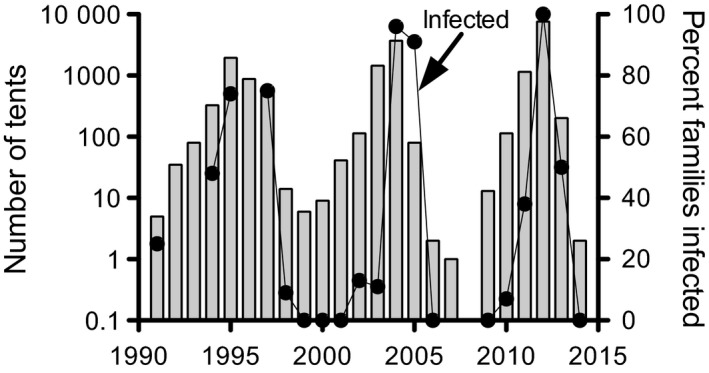
Change in numbers of tents (bars) for a western tent caterpillar population on Galiano Island, BC and the per cent families containing virus‐killed larvae (line) since 1990. The populations were too low in 2007 and 2008 to determine levels of infection.

**Figure 3:**
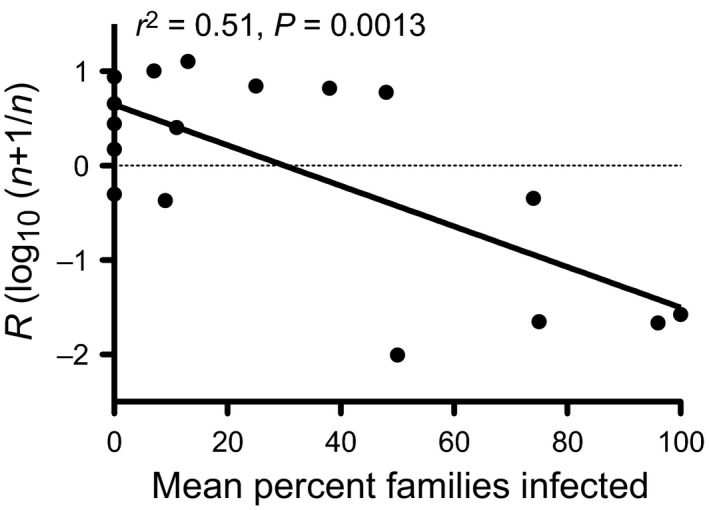
The relationship between the rate of population change [R] from 1 year to the next in one population of western tent caterpillars monitored on Galiano Island, and the level of infection for each year measured by the per cent of families with at least one NPV‐infected larva between the years 1991 and 2014 (J. H. Myers and J. S. Cory unpublished data).

Nucleopolyhedroviruses are DNA viruses that only infect the larval stage of Lepidoptera. After death, infected larvae release large numbers of virion‐containing occlusion bodies into the environment. Susceptible larvae become infected if they ingest occlusion bodies on foliage (Fig. 1). Viral infection increases rapidly with host numbers (Fig. 2). Populations decline following the viral epizootic, and viral infection is rarely detected in low‐density populations. To regulate population density, infection must be negatively related to the rate of population growth and this is the case (Fig. 3). Therefore, NPV infection has the potential to regulate populations of western tent caterpillars.

**Figure 4:**
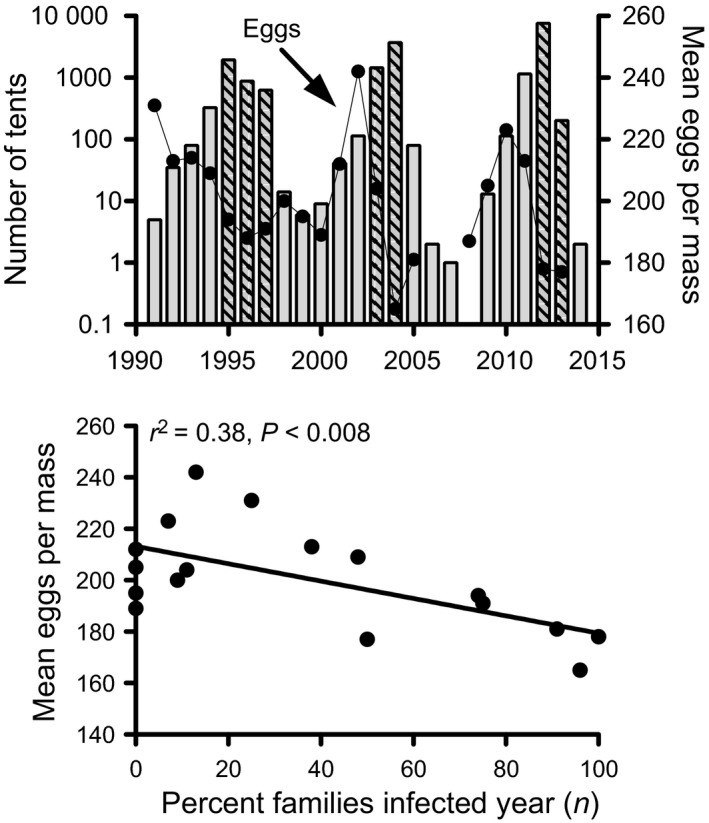
Top – the relationship between mean number of eggs per egg mass (fecundity) and numbers of tent caterpillar tents on Galiano Island. Bars are numbers of tents and circles are mean eggs per egg mass. Hatched bars indicate years of high viral infection. Bottom – the relationship between moth fecundity (mean number of eggs per egg mass) and the per cent families with viral infection for each year on Galiano Island.

While mortality from viral infection is a large part of the population dynamics of western tent caterpillars, a lag in recovery is required for cyclic dynamics. A factor that could provide this lag is reduced moth fecundity. Fecundity is often highest several years before the population peak (Myers and Kukan 1995; Myers 2000; Cory and Myers 2009), and continues to be low for several generations during the decline (Fig. 4). This reduced fecundity could be caused by maternal effects, a cost of resistance to infection (rejected in Cory and Myers 2009), reduced food quality and quantity (some support in Sarfraz et al. 2013), or could be related to virus through sublethal infection, or the costs associated with fighting infection. Many studies have found reduced size and fecundity of moths surviving exposure to NPV (review in Rothman and Myers 1996a,b; Rothman 1997; Myers et al. 2000; Cabodevilla et al. 2011). Comparing the trends of NPV infection to variation in the fecundity of moths shows that years of high numbers and high infection, 1995–1997, 2004 and 2005, and 2012 and 2013, produced moths with reduced fecundity (Fig. 4). Thus, the field data support the hypothesis that sublethal infection could be associated with the reduced fecundity of moths.

### Virus transmission and persistence

Baculovirus occlusion bodies can survive for considerable periods of time outside of their hosts if they are protected from ultraviolet irradiation. Thus, horizontal transmission from this persistent environmental reservoir is likely to be the main route of transmission when infection levels are high. Horizontal transmission is related to host density, and infection levels increase with rising host density above a theoretical threshold (although this can be altered by factors such as behaviour). As western tent caterpillars are gregarious, local density (within the tent) is always high and so the development of an epizootic will be based on the density of tents in the area. Manipulative studies have demonstrated two rounds of within‐family infection over the period of larval development (Rothman 1997).

Theoretical models often describe the transmission function using the mass action term (e.g. Anderson and May 1981) that assumes that the *per capita* transmission rate is density independent and should therefore remain constant over a range of host and pathogen densities. Field experiments on NPV transmission showed that this was not the case for pathogen density: the within‐family transmission coefficient decreased with increasing virus (cadaver) density (Beisner and Myers 1999). This has been observed in other studies (Fenton et al. 2002; see also below). This nonlinear relationship could be due to host limitation or heterogeneities in the system, such as varying susceptibility or spatial difference in infection risk (Dwyer et al. 1997; Fenton et al. 2002). The transmission coefficient did not vary with host density (family size) at the tree level, but the spread of virus between families was greater when families were larger (Beisner and Myers 1999) and larvae moved more.

Vertical transmission from parents to offspring can also occur with baculoviruses (Fig. 1) either through contamination of the outside of the egg (transovum transmission) or within the egg (transovarial transmission) (Cory 2015). In field and laboratory observations, 4–9% of egg masses from high‐density tent caterpillar populations yield infected larvae, whether the eggs are surface sterilized to remove contamination or not (J. H. Myers and J. S. Cory, unpublished data). Whether these infections are related to covert virus is unclear. NPV can be ‘stressed out’ into a virulent form through challenging larvae with a foreign virus. This occurred in forest tent caterpillars, *M. disstria*, challenged with the western tent caterpillar virus (Cooper et al. 2003a).

Covert (nonsymptomatic) infection, revealed using PCR, occurs in western tent caterpillar populations and was more common in the generation prior to a viral epizootic, that is the generation of moths that lays the eggs that yield larvae destined to die of virus at the beginning of the epizootic (J. S. Cory and J. H. Myers, unpublished data). Thus, the very rapid increase in infection levels as populations increase (Fig. 2) might be related to vertically transmitted virus. In low‐density populations, testing for covert infection is problematic from lack of samples, and we found none during population troughs.

### Genetic and resistance variation in the host

The maintenance of diversity in host and parasite populations remains one of the open questions in disease ecology (Lively et al. 2014). The fluctuating dynamics of tent caterpillars and their NPV could provide an excellent system to study selection on virus virulence and host resistance at different stages of the cycle (i.e. varying host density). The first stage in this process is to ascertain whether genetically based variation in phenotype occurs in host and virus populations.

Microsatellite markers indicated a high level of genetic variation within the tent caterpillar populations studied (Franklin et al. 2012, 2014), but no structuring among populations spread over a distance of approximately 100 km, and separated by stretches of water. This, and synchrony in the dynamics of different island populations (Cory and Myers 2009), suggests that considerable movement between populations occurs at high density. If some form of frequency‐dependent selection operated between host and virus genotypes, host genetic structure should differ between population peaks; however, we found no evidence to support this (Franklin et al. 2014). We also found no link between neutral genetic variation and disease resistance. Thus, even though populations go through severe bottlenecks every 5 to 8 generations, this does not create genetic variation among populations among sites or over time.

Genetic variation must translate into variation in disease susceptibility for pathogen epizootics to select for resistance or favour specific genotypes. We compared the susceptibility of different populations in the peak or prepeak year and then in two subsequent years of population decline. Families showed a large variation in resistance to NPV within each site, and some variation among sites (Cory and Myers 2009). One population that had not experienced a strong viral epizootic during the previous population decline was more susceptible to infection in the first two years of the study. Larvae from another population that experienced an early epizootic became significantly more resistant in the subsequent year. Thus, it appears that viral infection can select for increased resistance, but this does not stop the epizootic.

### Genetic variation in the virus population

Baculoviruses are often highly variable genetically. The maintenance of this variation in NPVs is thought to be facilitated, in part, by their unique morphology whereby multiple infective genomes are packaged together in virus particles, which are themselves occluded within the proteinaceous occlusion bodies (Clem and Passarelli 2013). McplNPV diversity in low‐density host populations was surprisingly high. The spatial structure of the virus was also hierarchical, with the virus from families and then populations being more similar than those on different islands (Cooper et al. 2003b). This is likely to be an underestimate of the variation within the virus population. At high host densities, when virus infection is widespread, mixing of virus between families could provide the opportunity for virus recombination. Thus, a key issue here is whether pathogen diversity changes at different stages in the population cycle and whether this influences virulence, and potentially changes in host resistance.

### Maintenance of variation and local adaptation

How is virus variation maintained? If one pathogen isolate was highly virulent or host genotype broadly resistant (i.e. had superior fitness), we would expect them to dominate the populations. But this is not the case. Various possibilities have been suggested for the maintenance of pathogen variation, including trade‐offs between virus traits, differential selection on virus genotypes, such as via environmental variation, and facilitation in mixed infections (see Hodgson et al. 2001). Initial studies addressed environmental factors: the interaction with host plant.

The plant fed on by herbivorous insects often alters host susceptibility to entomopathogens due to interactions within the mid‐gut and impacts on resistance (Cory and Hoover 2006). In addition, baculoviruses can persist outside their hosts and larvae often acquire their infections from this external reservoir. Thus, the virus has a direct interaction with foliar chemicals when it is ingested and the virus can have a prolonged relationship with the host plant through environmental persistence. A study of another lepidopteran species, *Panolis flammea*, demonstrated that host plant can differentially impact the pathogenicity and productivity of different baculovirus variants (Hodgson et al. 2002). We therefore hypothesized that a pathogen could adapt to a locally dominant host plant.

Comparison of virus isolates from three geographically distinct sites with different dominant host plants demonstrated that in two populations, the speed of kill was fastest on the dominant food plant of the tent caterpillar population at that site: red alder or wild rose (Cory and Myers 2004). This association did not hold for the third population for which the dominant host plant was apple (*Malus domestica*). This latter population also goes extinct between outbreaks and thus depends on immigration to recolonize. Thus, local adaptation to the host plant would not be expected. This illustrates the potential for environmental factors to modulate host–pathogen relationships and provide differential selection on genotypes, as well as demonstrating that complex three‐way interactions can influence evolutionary adaptation (Biere and Tack 2013).

## African Armyworms

While many of the most obvious pathogen epizootics that have been recorded are in temperate forest species, other groups of Lepidoptera in very different environments often support high levels of endemic baculovirus infection which can develop into large‐scale epizootics; these are the armyworms. The term armyworm has been applied to many species of Lepidoptera often within the genera *Spodoptera* and *Mythimna* in the Family Noctuidae. Armyworms are characterized by a tendency to form large groups that move *en masse*, eating any suitable foliage in their path. Many armyworm species also migrate large distances, including between continents. One interesting feature of armyworms is their propensity to die from baculovirus epizootics, even in tropical regions. For example, NPV‐induced mortality of *Spodoptera exigua* on tomatoes in Mexico was reported as ranging from 15% to 90% at its peak in different populations (Alvarado‐Rodriguez 1987). Similarly, prevalence of NPV in *S. frugiperda* in Louisiana, USA, varied from 53% to 60% depending on host plant (Fuxa 1982; see Fuxa 2004 for a review). The high prevalence of baculoviruses in tropical and subtropical regions where ultraviolet light will inactivate the virus is surprising. In addition, the migratory behaviour of many of the populations has the potential to remove them from virus that might persist in the environment.

### Population outbreaks, host polyphenism and virus variation

**Figure 5:**
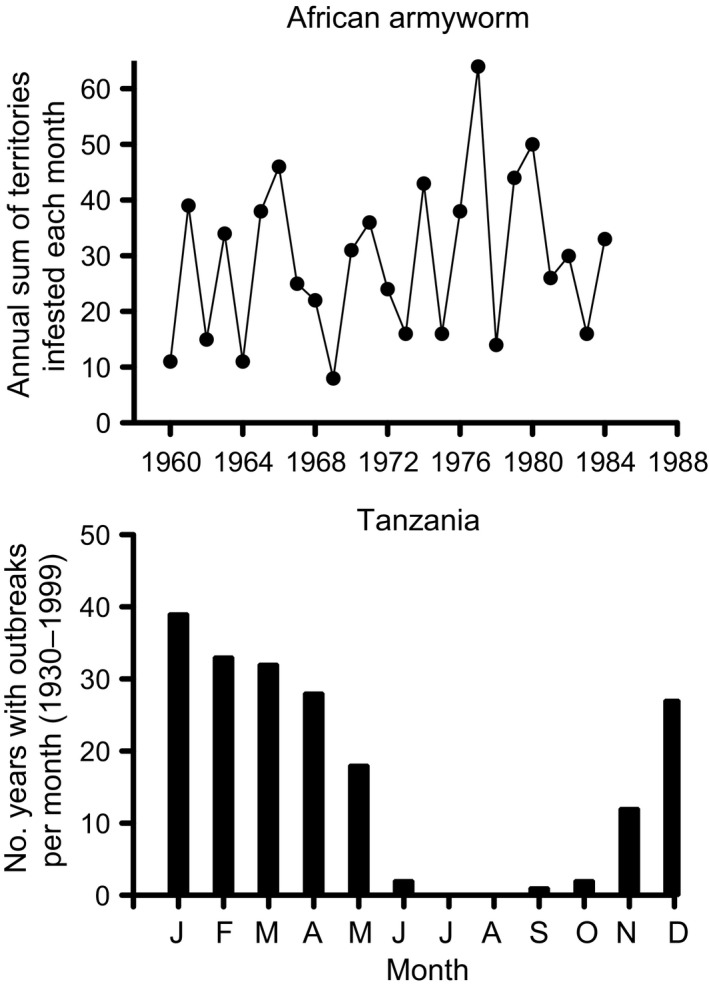
Top – The annual sum of the number of territories recording larval infestations of *S. exempta* in East Africa from 1940 to 1984. Data summarized from Haggis (1986). Bottom – Number of years with recorded outbreaks of *S. exempta* in Tanzania summed on a monthly basis for 1930 to 1999 (from table 1 in Rose et al. (2000).

A particularly interesting species is the African armyworm, *Spodoptera exempta*. This species occurs mainly, but not exclusively, in East Africa and can be a devastating pest on a wide range of graminaceous crops and natural grasslands. Larval densities can reach up to 1000 larvae/m^2^ (E. Redman pers. comm.). Monitoring and forecasting the frequency of highly damaging outbreaks of *S. exempta* was initiated by Haggis in 1940 using a combination of light traps for the adults and reports of larval infestations, and outbreaks have been monitored until recently (Haggis 1986; Rose et al. 2000) (Fig. 5).

These studies indicated that populations of African armyworm are influenced by rainfall and low‐density, source populations can persist in coastal and highland areas of East Africa where rainfall is more frequent (Rose et al. 1995). Outbreaks are strongly influenced by precipitation during the rainy season (March–May), and occur as a result of wind convergence, followed by wind‐assisted migration from outbreak areas (Rose et al. 2000; Dewhurst et al. 2001). A key feature of this species is phase polyphenism, and it has a dark, gregarious larval phenotype and a lighter green, solitary larval form. The gregarious form causes the major outbreaks, but populations persist as the low‐density morph, and are very hard to locate.

An NPV was isolated and identified from *S. exempta* after surveys in the early 1960s (Brown and Swaine 1965). Manipulative experiments and field studies clearly indicated that NPV was the major mortality factor in high‐density populations and was positively associated with rainfall and negatively associated with sunshine (Persson 1981; Odindo 1983). There were also indications that mortality was influenced by the host plant that the larvae fed on with lower levels on the stargrass (*Cynodon dactylon*) than on maize (Persson 1981). *Spodoptera exempta* can have 6–8 generations during the outbreak season, and NPV infection levels tend to rise as the season progresses, with infection in some outbreaks approaching 100% (Rose et al. 2000; Chapman et al. 2015). The potential use of this virus as a control agent was investigated more recently (Grzywacz et al. 2008); however, the ecology and scale of this system and the sporadic nature of the outbreaks make it a challenging target. In common with other baculoviruses, *S. exempta* NPV (SpexNPV) is highly variable genetically (Redman et al. 2010) and phenotypically (E. M. Redman, K. Wilson and J. S. Cory, unpublished data). More interestingly, virulence increases with strain diversity (E. Redman and J. S. Cory, unpublished data). This ties in with earlier work on another lepidopteran system that showed that a combination of two baculovirus strains was more pathogenic than the single isolates (Hodgson et al. 2004). The mechanism by which this occurs remains to be elucidated; however; it could have major implications for both practical pest management and the evolution of resistance (Cory and Franklin 2012).

Earlier studies on other lepidopteran species had demonstrated that solitary larvae are more susceptible to viral infection than those reared at high density (Kunimi and Yamada 1990; Goulson and Cory 1995). This also occurs in *S. exempta* (Reeson et al. 1998). Thus, in manipulative field experiments, virus mortality and the transmission parameter were lower for larvae reared gregariously (Reeson et al. 2000). This effect has been termed density‐dependent prophylaxis, and is suggested to be a response to rising host densities, and thus increasing disease risk, whereby increased resources are allocated to disease resistance (Wilson and Reeson 1998). Density‐dependent prophylaxis has, however, not been found in all Lepidoptera. For example, a negative relationship between host susceptibility and baculovirus infection occurred in the gypsy moth, *Lymantria dispar* (Reilly and Hajek 2008). Density‐dependent prophylaxis may be limited to Lepidoptera with phase polyphenism.

### Viral persistence

The presence of such high levels of mortality due to a pathogen like a baculovirus, which primarily relies on horizontal transmission and the persistence of virus occlusion bodies when the host dies, is perhaps surprising in a migratory, tropical species as it is not clear how the virus persists in the system. SpexNPV is host specific, and alternative hosts are therefore not an option. Environmental persistence or direct contact with virus‐infected cadavers resulting in horizontal transmission (Fig. 1) will occur within populations at high density. It seems unlikely, however, to explain how the virus survives through the solitary stage and when the insect does not reoccur in the same area for many years. As with the tent caterpillars at low density, a potential mechanism for persistence is vertical transmission with the migrating adult moths carrying the virus (Fig. 1).

Early studies indicated that SpexNPV could be passed from female moths to their offspring within the egg, that is, not through external contamination (Swaine 1966). We extended these studies by comparing vertical transmission in the two larval morphs and using PCR to identify both viral DNA and RNA. Larvae reared solitarily or in groups in the laboratory both transmitted overt virus infection to their offspring that then died, but this was greater in insects reared solitarily (85% infected vs 40% for gregarious) and was enhanced if the insects were exposed to virus in the parental generation (80% infected vs 45% for unexposed controls). This clearly illustrates that horizontal transmission in one generation can impact vertical transmission to the next (Vilaplana et al. 2008). Virus could be transmitted equally well by both sexes and was not affected by surface sterilization of the eggs, confirming that the virus was transmitted within the egg. However, there were other differential costs to virus exposure. Pupae from gregarious insects weighed less after virus exposure, but those from solitary insects, which weigh more initially, did not weigh less after exposure (Vilaplana et al. 2008). This could affect the dynamics of the armyworm although under laboratory conditions, this did not translate into significant differences in fecundity. Interestingly, virtually all adult insects captured in the field from an outbreak population were positive for covert NPV. This does not usually result in active infection in the offspring, and this level of covert infection was not lost after being reared for multiple generations in the laboratory (Vilaplana et al. 2010; Graham et al. 2012). Thus, the virus was carried vertically without cost, at least within the relatively benign laboratory conditions.

It has long been thought that Lepidoptera can carry ‘latent’ (covert) baculovirus infections that will spontaneously activate after some type of triggering event. PCR studies have indicated that there is some level of transcription going on within the adult insect, so these infections are probably more accurately termed ‘persistent’. However, it has proved difficult to identify a consistent trigger; infection with other viruses (see tent caterpillar section), food plant and various stresses have all been implicated as triggers, but whether they play an important role in the initiation of virus epizootics is not known.

Understanding how mixed mode transmission evolves and when a particular transmission strategy will predominate is important for disease management, as well as in the use of entomopathogens as pest control agents. It might be expected that vertical transmission would evolve to cope with situations of prolonged host absence or poor environmental persistence (Cory 2015). Many of the species in which vertical transmission of overt disease occurs at relatively high levels are migratory species or species with more cryptic lifestyles (Kukan 1999), but the link to covert infection remains unclear.

Theoretical examination of the conditions under which covert infection might evolve suggests that it is more likely where there are seasonal fluctuations in host density (which fits the examples discussed here) or if covert infection is protective (Sorrell et al. 2009) as might be expected in true symbionts (Jones et al. 2011). There is little support for a protective role for covert infections in baculoviruses, and these models predict a much lower level of covert infection than that demonstrated in *S. exempta*. Thus, it is clear that other mechanisms are involved.

### Disease and migration

From the host perspective, the link between migratory behaviour and disease has been examined in detail in relation to monarch butterflies. Escape from the pathogen and ‘migratory culling’ (loss of insects due to disease) have both been suggested as possible drivers for insect migration (Altizer et al. 2011; Hall et al. 2014). Various theoretical relationships between migratory capacity and pathogen load have been suggested based on possible trade‐offs between flight and immune function (Chapman et al. 2015). It is not clear how retaining a covert baculovirus infection might impact the migratory capacity of *S. exempta* (or any other species). However, unless an adult can be infected by a replicating pathogen, a direct effect of the pathogen is unlikely, and adults are most likely to be passive vectors of the pathogen. The impact on migrating insects is more likely to be a reflection of pathogen exposure in the larval period (sublethal effects, Rothman and Myers 1996a), primarily through reduced adult size and condition and inadequate investment in the structures and resources necessary for the migratory flight, rather than pathogen load.

## Gypsy moth in North America

Tussock moths (Subfamily Lymantriinae) are another group of insects that are susceptible to pathogen epizootics, particularly those involving baculoviruses. Disease often occurs in species in the genera *Orgyia*,* Lymantria*,* Euproctis* and *Leucoma*, and epizootics have been recorded in these species in temperate northern forests since the late 19th century. Gypsy moth, *Lymantria dispar,* is perhaps the best studied of these species, primarily because it is an invasive species in North America, originally introduced in the late 1860s. The battle against gypsy moth as an invasive species is a fascinating story in biological control. Over 90 years, 55 species of natural enemies were released in this programme (review in Hajek et al. 2007). Gypsy moth is a highly polyphagous species, which has one generation a year. It overwinters in the egg stage and a female lays her eggs as a single mass. The females in the European/North American gypsy moth are flightless (although Asian gypsy moth females fly). However, early instar larvae can disperse by ballooning on long silken threads.

### Baculoviruses, pathogen transmission and tritrophic effects

**Figure 6:**
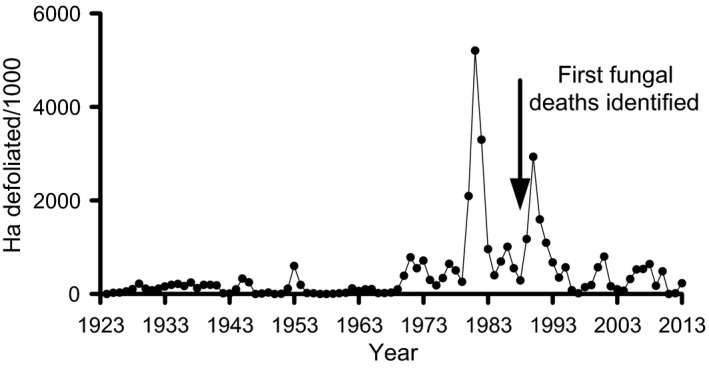
Gypsy moth populations monitored annually as the area defoliated in north‐eastern USA by the US Forest Service Northeastern Area (http://www.na.fs.fed.us/fhp/gm/cfm_files/dsp/dsp_defchart.cfm). Arrow indicates the year when mortality from fungus was first observed.

Gypsy moth dynamics are characterized by periods when the populations exist at low densities, with rapid outbreaks building up over several years, followed by population collapse, often due to pathogens, particularly LdMNPV (Elkinton and Liebhold 1990). Two periods of major outbreak occurred in north‐eastern USA in 1981 and 1991, as seen in defoliation data collected by the US Forest Service (Fig. 6). In addition, subharmonic peaks have occurred at approximately 5‐year intervals since the late 1960s (Bjørnstad et al. 2010). No major outbreaks in this region have occurred in the 25 years since 1991, immediately after the invasion of a fungal pathogen, although gypsy moth continues to spread to the south and west.

It is thought that LdMNPV was introduced with parasitoids. It might be expected that the virus would have little variability if there was only a limited introduction, although North American LdMNPV isolates show some variation and have different phenotypes (Shapiro et al. 1984). Given the propensity for recombination in MNPVs, the generation of multiple new recombinants since introduction is highly likely. Recent genetic analysis showed that the Asian LdMNPVs are distinct from those found in Europe and North America (Harrison et al. 2014).

The initial infection of larvae occurs when the neonates feed on the chorion of the eggs, resulting in an early wave of NPV infection. The death of these larvae initiates a second wave of infection in the late instars (Woods and Elkinton 1987). The virus on the eggs appears to result from contamination from the bark on which they were laid, rather than from the mother, and can be removed by surface sterilization (Elkinton and Liebhold 1990). Whether LdMNPV can be transmitted vertically in an overt or covert form is less clear. A low level of vertical transmission occurred, however, in several egg masses of sublethally infected females in a laboratory study (Myers et al. 2000).

Perhaps the biggest contribution of gypsy moth–NPV studies (together with studies of other baculovirus systems, see Tompkins et al. 2011) is in the estimation and understanding of pathogen transmission. A straightforward experimental procedure combined with simple differential equation models was used to estimate the transmission parameter and predicted within season virus dynamics relatively successfully in high‐density gypsy moth populations (Woods and Elkinton 1987; Dwyer and Elkinton 1993). The fit of the model to low‐density populations, which had higher‐than‐expected mortality, was later improved by adding a term to account for heterogeneity in susceptibility (Dwyer et al. 1997). Similar experimental approaches were used to examine the underlying assumptions of many host–pathogen models, for example mass action, and demonstrated that virus transmission varies nonlinearly with increasing pathogen and host density (D'Amico et al. 1996; Dwyer et al. 1997). These population‐scale experiments on pathogen transmission have been incorporated into models of longer term gypsy moth dynamics. More recent studies have examined the impact of other factors such as host behaviour and host evolution on risk of infection and/or host population dynamics (e.g. Elderd et al. 2008; Parker et al. 2010). These population models often show a good fit to the 1981 and 1991 outbreaks based on defoliation, but not to the subsequent more frequent and less dramatic fluctuations (Fig. 6) (but see Bjørnstad et al. 2010). Although these results have been interpreted to indicate a role for LdMNPV in gypsy moth dynamics, testing these relationships is more difficult.

One particularly interesting area in the gypsy moth–NPV interaction, as in the tent caterpillars, is the impact of host plants. In common with many baculovirus–lepidopteran relationships, the susceptibility of gypsy moth to NPV can be altered by the larval host plant. For example, infection levels are lower on oaks than aspen (Keating et al. 1988). More intriguing is the impact of induced defences; early studies indicated that induction of tannins as a result of gypsy moth defoliation reduced NPV infection, as well as fecundity (Hunter and Schultz 1993). Models suggested that these changes would be destabilizing at intermediate tannin levels (Foster et al. 1992). Later field studies refuted this as they found that large changes in tannin levels did not occur sufficiently early in the season to affect the gypsy moth–NPV interactions significantly (D'Amico et al. 1998). In addition, experimental and modelling studies by Dwyer et al. (2005) using two species of oak demonstrated how behavioural changes of larvae can compensate for foliage‐based differences in susceptibility. In general, they found that host plant effects were weak and likely to be swamped by the impact of high doses of virus. They strongly suggested that two species rather than tritrophic models were sufficient to describe gypsy moth dynamics. More recently, however, a role for induced plant defences in gypsy moth outbreaks has again been suggested based on variation in forest composition and proportion of inducible oak trees (Elderd et al. 2013). More quantitative information is needed on the actual population and disease dynamics of gypsy moth and the distribution and composition of different forest types, for the relevance of these models to be interpreted correctly.

### Fungal infection

In 1989, scientists observed gypsy moth cadavers hanging in trees in forests in north‐eastern USA. The responsible pathogen was identified as the fungus *Entomophaga maimaiga* (Andreadis and Weseloh 1990). Although previous introductions of fungus had been made in 1910 and 1911 from Japan, where it was reported to cause epizootics, it was speculated that this epizootic was caused by a new strain that had been accidentally introduced to North America (Hajek et al. 1990, 1995; Weseloh 1998). Genetic analysis showed that North American isolates of the fungus were less diverse than those in its native range in Japan, as might be expected if the fungus was the product of a small number of introductions (Nielsen et al. 2005a). The US isolates were most closely related to those in Japan, indicating that this was the likely source, although they formed a distinct group. Bioassays also showed that some of the isolates differed in their speed of kill and the type of spores produced (Nielsen et al. 2005b).

Entomopathogenic fungi differ from most other groups of insect pathogens in that they do not need to be ingested to initiate infection; they invade through the host's cuticle. In addition, an interesting characteristic of *E. maimaiga* is that it has two forms: overwintering resting spores and conidia that are actively released from the cadavers. Resting spores can persist for over a year: they produce a germ conidium and are thought to initiate the first round of infection in neonate larvae. Infections from germ conidia only result in conidia, and resting spores tend to be produced in the later larval instars (Hajek 1999; Reilly et al. 2014). These processes require high humidity or high moisture levels. The resting spores provide the main persistence mechanism for *E. maimaiga* (Hajek et al. 2004) that has a very narrow host range and no vertical transmission. The prevalence and spread of *E. maimaiga* have been well documented as its discovery and its very different life‐history characteristics provide an interesting contrast to LdMNPV. How it interacts with LdMNPV and its influence on gypsy moth population dynamics, which theoretical models suggest are primarily driven by LdMNPV, are key issues.

Recent studies have recorded fungus‐killed larvae in populations along the western edge of the gypsy moth invasion front where population density tends to be low. Over three years, average levels of fungal mortality ranged from 16% to 46% while observed infection by NPV was less than 3% of larvae (Hajek and Tobin 2011). Prevalence of both pathogens was positively related to gypsy moth density, although the fungus tended to be found in lower density populations. This is in contrast with other studies that suggest that *E. maimaiga* is density independent (Hajek 1999; Liebhold et al. 2013). Thus it would appear that *E. maimaiga* transmission is to some extent condition dependent. In addition, fungal prevalence was strongly influenced by rainfall and negatively affected by temperature, whereas LdMNPV was only influenced positively by increasing temperature (Hajek and Tobin 2011). Liebhold et al. (2013), in a comparison of *L. dispar* dynamics before and after *E. maimaiga* introduction, found that *E. maimaiga* usually tends to cause higher mortality than the virus. However, it did not affect the strong density dependence of the viral pathogen, which is interesting as host populations should have been lower if fungal deaths occurred. The fungus appears to be playing an increasingly large role in areas where gypsy moth is established.

Gypsy moth populations declined abruptly in the mid‐Atlantic states of the USA in 2009, and fungal infection was the dominant mortality factor (Hajek et al. 2015). If fungal mortality maintains gypsy moth at low densities over time, population outbreaks and cyclic dynamics should be reduced (see further discussion in Myers and Cory 2013). It is surprising that an analysis by Allstadt et al. (2013) showing a transition from approximately ten‐year to five‐year population cycles or noncyclic dynamics of gypsy moth in north‐eastern USA (see Fig. 6) did not even discuss the potential impact of fungal infection.

## Monarch butterflies

The monarch butterfly, *Danaus plexippus,* is an iconic species in North America that is well known for its annual migrations between breeding sites in eastern North America, and overwintering sites in Mexico, or between upland breeding to coastal overwintering areas in western North America (Urquhart 1960; Brower et al. 2002). Chemicals sequestered from milkweed cause the butterflies to be distasteful to potential bird predators and they serve as models in mimicry systems. Monarchs have multiple generations per year as populations migrate north. Females may mate several times. They lay eggs individually on milkweed plants and larvae can be cannibalistic. Female butterflies in the last generation of the season do not mate and make the migration back to overwintering sites. They will mate the next spring before starting their northern flight.

In 1970, a neogregarine parasite *Ophryocystis elektroscirrha* (OE) (McLaughlin and Myers 1970) was discovered that attacks monarchs and their congener, the Florida Queen butterfly, *D. gilippus berenice*. Currently, monarchs are under threat from various directions; trees in its overwintering sites in Mexico are being logged, storms have devastating impacts on over‐wintering populations, and high herbicide use in the Midwest of North America is depleting their milkweed food plants (Flockhart et al. 2014). Infection by OE could be an additional threat.

### Impact of *Ophryocystis elektroscirrha*

*Ophryocystis elektroscirrha* replicates both asexually and sexually within the host larvae to produce a large number of spores that are released on the cuticle of the adult butterflies, primarily on the abdomen, when they emerge from the pupa. It is interesting that although the parasite replicates in the larvae, the impact is primarily on the adults. Adults heavily infected as larvae can have difficulty in expanding their wings and die, while less infected individuals have reduced longevity and reproduction (Altizer and Oberhauser 1999; de Roode et al. 2009). Butterflies with low levels of infection as larvae appear to be unaffected (McLaughlin and Myers 1970). A distinct advantage for studies of this parasite is that the spores can be readily seen on infected butterflies and can be sampled and counted on field‐collected individuals even by nonspecialists.

### Transmission

**Figure 7:**
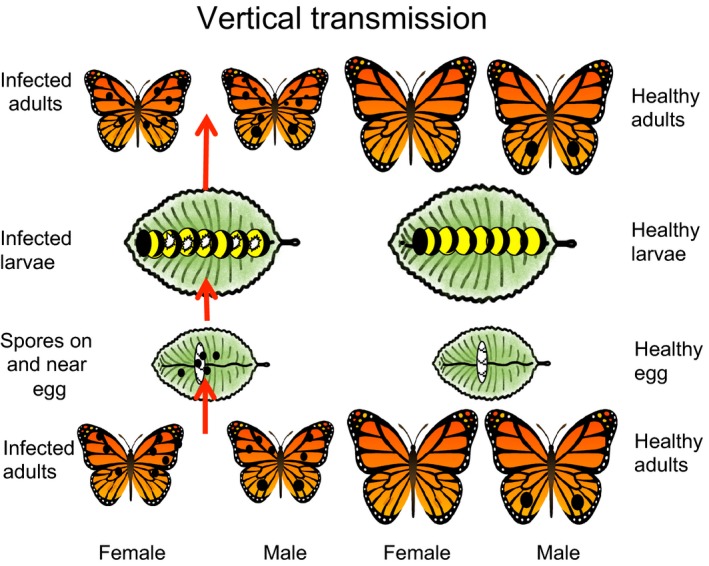
The neogregarine parasite of monarch butterflies reproduces in the larvae. Adults developing from infected larvae are contaminated by spores on their bodies, particularly their abdomens, following emergence from the pupa. The parasite is transferred vertically from adult females to their eggs or larval feeding sites at oviposition. At hatching, larvae ingest spores that reproduce through the larval stage (indicated by white dots above), and then form spores that contaminate adults at emergence. Adults arising from infected larvae can be smaller and have reduced longevity compared to healthy individuals. Horizontal transmission will occur if spores are ingested by larvae that are not offspring of the ovipositing female. Transmission from males to offspring is low.

Spore‐contaminated adults scatter spores on larval food plants and females contaminate eggs (male–female transfer of spores in mating is limited, de Roode et al. 2009). A single‐spore‐infected larva can result in 10^5^ to 10^7^ spores on the adult (de Roode et al. 2007). Larvae ingest the spores on hatching or while feeding on plant leaves (Fig. 7). There is no horizontal transmission between larvae. Longevity is negatively related to increasing spore load, and higher spore load is related to greater transmission to eggs. The distribution of spores can be limited, however, if butterfly survival and flight are reduced for highly infected individuals (de Roode et al. 2009).

### Variation, interactions and trade‐offs

The Monarch *– O. elektroscirrha* system lends itself to experimental manipulation and has become a model for the study of invertebrate–parasite interactions and exploration of some of the assumptions and theory underlying disease ecology and evolution. For example, different parasite isolates vary in virulence and a positive relationship was found between virulence (adult life span) and transmission (spore load) over a range of isolates. This is consistent with one of the assumptions underlying evolution of virulence theory (de Roode and Altizer 2010). However, these relationships were affected by genotype X genotype interactions. Western populations of monarchs from California and those from eastern populations do not differ in their resistance to OE, but western parasites are more virulent than eastern parasites (de Roode and Altizer 2010). The proposed trade‐off between the costs (virulence) and benefits (transmission) of increased parasite replication underpins many evolutionary studies. In the monarch butterfly, de Roode et al. (2008b) demonstrated this trade‐off: OE fitness was maximized at intermediate levels of replication. When levels of parasite replication are too high, transmission is reduced because the probability of emergence of highly infected female monarchs is low.

### Tritrophic interactions

The role of host plant has already been shown to influence the virulence of several groups of entomopathogens; however, impact on protozoans had not received any attention. In the baculovirus studies discussed above, all the hosts were highly polyphagous. In contrast, monarch butterflies only feed on milkweed; thus, they might be expected to be more adapted to the chemistry of this specific host group. Milkweed species vary in the levels of cardenolides that they produce. A native species of milkweed that occurs on the eastern migration route, *Asclepias incarnata,* has lower levels of cardenolides than an introduced species, *A. curassavica,* which is more common in Florida. Adults resulting from larvae fed *A. incarnata* in laboratory trials had higher parasite loads and reduced longevity, compared to those fed *A. curassavica* (de Roode et al. 2008a). Although the mechanism behind this is not clear, it suggests a strong protective impact of cardenolides, and in this study, the impact of host plant outweighed the effect of variation between parasite genotypes. Unlike baculoviruses, where challenged larvae can potentially ‘self‐medicate’ and offset the costs of resistance by feeding on more protein (Lee et al. 2006); eating high‐cardenolide plants after OE infection appears to have little impact on infection or spore production (de Roode et al. 2011). However, females that were exposed to the parasite as larvae preferentially laid their eggs on the species with the higher level of cardenolides (Lefèvre et al. 2010). Interestingly, nonmigratory monarch populations have higher OE infection levels in the southern USA where *A. curassavica* is common (Satterfield et al. 2015). This is likely associated with the higher butterfly densities at these southern sites that allow parasite populations to increase.

Sternberg et al. (2012), in a more extensive study of 12 different milkweed species, showed that larval host plant species influenced the size and longevity of resulting adults of uninfected as well as infected larvae. For challenged larvae, both their tolerance, as indicated by the relationship between the longevity and number of spores on resulting butterflies, and their resistance (the probability of becoming infected) varied with the species of the milkweed ingested by the larvae (Sternberg et al. 2012). Resistance and tolerance have very different evolutionary trajectories as resistance acts to limit parasite growth, whereas tolerance mechanisms are more to do with reducing fitness costs. This study suggests that this relationship can be modulated by environmental factors.

### Migration and disease

Migration success of both male and female butterflies is related to the level of parasitism. Altizer et al. (2000) suggest that the longer migration of eastern monarchs selects against highly parasitized individuals. This should affect parasite transmission via mating as females delay mating until the end of migration. Heavily infected males migrate shorter distances than uninfected males and thus will not make the return to the overwintering sites and are not available for mating and spore transfer there. Bartel et al. (2011) found that the prevalence of parasites among migrating butterflies was less at more southern locations and at the overwintering sites. This supports the ‘migration culling’ hypothesis and is likely to reduce the long‐term build up of OE. Levels of parasitism in the breeding sites also decreased as the season progressed, supporting the idea of ‘migratory escape’. A theoretical analysis also indicated that both longer migrations and less time at the breeding site, and migration reducing the survival of infected individuals, reduced the probability of the pathogen invading the population (Hall et al. 2014).

Levels of parasitism by OE in eastern migratory monarchs tend to be low and stable. Altizer et al. (2000) tracked levels of parasitism between 1966 and 1996 and consistently found less than 8% of individuals with high levels of spores. For the western migratory population monitored in four years between 1978 and 1996, 30% to 40% of individuals had a high prevalence of spores. For the nonmigratory population of monarchs monitored in four years between 1990 and 1996 in Florida, 75% to 90% of individuals had high spore levels (see also Satterfield et al. 2015). Higher levels of infection by OE in some nonmigratory populations support the theoretical model, but this is not always the case. On four different islands in Hawaii, nonmigratory populations had levels of infection (estimated from the proportion of individuals with high numbers of spores) varying from 4% to 85% (Pierce et al. 2014). Thus, other factors than just continued use of a location are influencing parasite infection. These populations varied in their food plant species but were genetically similar which may indicate that movement among populations is common.

**Figure 8:**
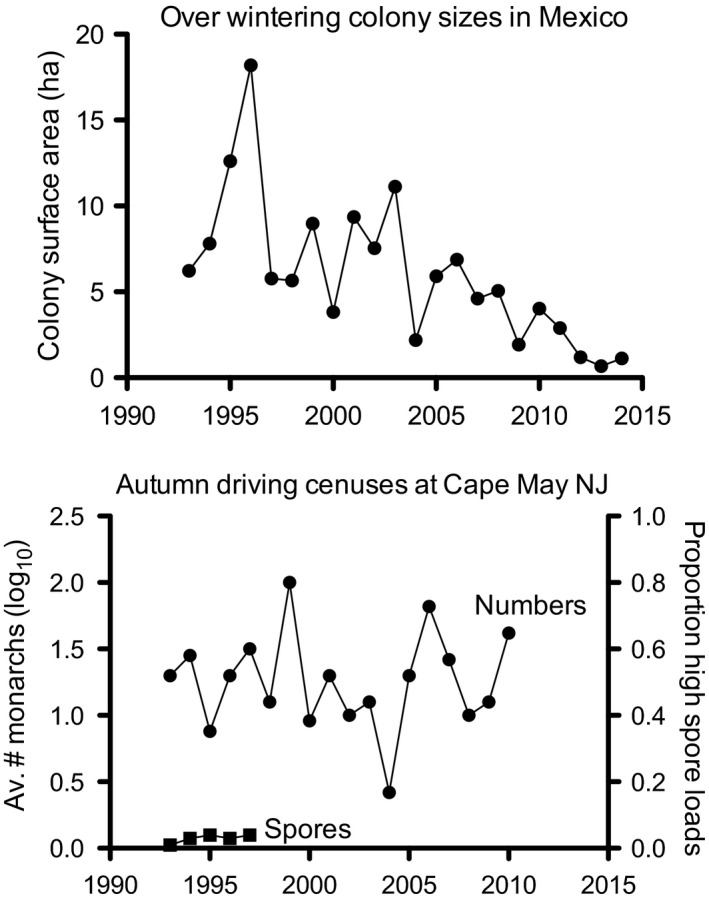
Top. Monarch populations are estimated over time by the area of overwintering butterfly colonies (data from Vidal and Rendón‐Salinas 2014). Bottom. Total counts of butterflies made in driving censuses at Cape May NJ (circles – data from Davis 2012) and the proportion of individuals with high spore loads (squares) recorded for Eastern migratory populations by Altizer et al. (2000).

The role of this parasite in host population dynamics may fade in importance compared to influences of food plant abundance and overwintering habitat availability. Population trends for monarch butterflies over time show two different patterns (Fig. 8). The sizes of monarch colonies overwintering in Mexico have been declining since the late 1990s. In contrast, counts of migrating monarchs at Cape May NJ varied, but showed no downward trend between 1993 and 2011. If the levels of infection by OE in migratory populations have continued at less than the 8% reported between 1966 and 1996 (Altizer et al. 2000), there would appear to be no association between population levels and OE infection. While definitive studies have not been carried out, it is unlikely that parasitism has had a strong impact on the decline in numbers of overwintering monarchs.

## Conclusions and future directions

Insects and their pathogens are increasingly being used to address fundamental questions in disease ecology and evolution. For example, baculoviruses have been developed as a system to explore the factors that are important in pathogen transmission and assumptions underlying different theoretical models. All the systems discussed illustrate the importance of environmental factors in modulating virulence, resistance and the risk of infection. In particular, the impact of larval food plant in interactions between tent caterpillars, gypsy moths and monarch butterflies clearly demonstrates that the virulence and pathogenicity of a micro‐organism can be highly context dependent.

Insect migration and population fluctuations of the host strongly influence the persistence and transmission strategies of pathogens. In the absence of alternative hosts, vertical transmission as either an overt infection or a covert form of virus, or an environmentally persistent life stage, such as a protected occlusion body (NPV) or spore (fungus or OE) is necessary for persistence of pathogens between host outbreaks. In several of the pathogens described here, a mix of vertical and horizontal transmission occurs and so there is clearly benefit to this. While a good theoretical framework for the evolution of various types of vertical and mixed mode transmission exists, experimental studies to test these ideas, particularly those involving viruses, are challenging.

The widespread genetic diversity within many insect and pathogen populations makes them powerful systems to test theories relating to the maintenance of diversity and its role in disease spread, key issues highlighted by Lively et al. (2014). Some of the work described here has already started to address the potential factors that could influence the maintenance of diversity in host and pathogen populations, such as differential selection and benefits of multiple genotypes infections.

Even in these well‐studied systems, however, it remains clear that long‐term studies on pathogen dynamics and the role of pathogens in insect populations and communities in the field are extremely limited. Pathogens can be density dependent, regulating factors, and this may require pathogens that are highly virulent with horizontal transmission between infected larvae upon death. Whether a pathogen that develops in the larval stage but is transmitted by adults can act in the same density‐dependent manner remains to be tested. Pathogens can also act in a density‐independent manner and limit host densities.

Providing convincing evidence for the role of pathogens in insect populations remains challenging. Long‐term population data are the key to demonstrating the relationship between NPV and population growth in the tent caterpillars. For the other systems described, population data are based on surrogates such as numbers of outbreaks, areas defoliated and areas of overwintering sites. Thus, mechanisms related to population processes are difficult to impossible to demonstrate.

The revolution in genomic techniques should make future study of insect–pathogen dynamics in natural systems more tractable. In addition, insects and their pathogens will continue to provide a wealth of opportunities to explore the processes underlying disease ecology and evolution. Although models and theoretical explorations of pathogen and host interactions will continue to contribute to this field, it must be kept in mind that the heterogeneity of field situations will have a strong role in determining outcomes. Tests of predictions based on field studies are crucial to understanding the roles of pathogens and their hosts.
